# EPIGENETIC AGE AND MUSCLE CHARACTERISTICS IN BALTIMORE LONGITUDINAL STUDY OF AGING PARTICIPANTS

**DOI:** 10.1093/geroni/igad104.0584

**Published:** 2023-12-21

**Authors:** Ann Zenobia Moore, Pei-Lun Kuo, Qi Yang, Bennett Landman, Eleanor Simonsick, Luigi Ferrucci

**Affiliations:** National Institute on Aging, National Institutes of Health, Baltimore, Maryland, United States; National Institute on Aging, National Institutes of Health, Baltimore, Maryland, United States; Vanderbilt University, Nashville, Tennessee, United States; Vanderbilt University, Nashville, Tennessee, United States; National Institute on Aging, National Institutes of Health, Baltimore, Maryland, United States; National Institute on Aging, National Institutes of Health, Baltimore, Maryland, United States

## Abstract

Changes in muscle, including loss of muscle mass and fat infiltration, contribute to loss of muscle strength and subsequent declines in physical function in later life. Measures of body composition are consistently associated with DNA methylation (DNAm) in cross sectional studies. Whether differences in DNAm are informative with respect to longitudinal muscle characteristics has not been fully explored. Here we evaluate the relationship between epigenetic age metrics and computed tomography (CT) derived measures, including areas of muscle, subcutaneous fat, and intermuscular fat, as well as the mean and standard deviation of muscle intensity at mid-femur, in the Baltimore Longitudinal Study of Aging. Participants were at least 50 years old at the evaluation of DNAm (n = 471, 51% female), 61.8% of participants had three or more CT scans and mean follow up time was 6.0 years. Relationships between epigenetic age metrics including Horvath and Hannum DNAm age, PhenoAge, GrimAge and DunedinPACE at the index visit, and the trajectory of muscle composition were evaluated using multivariable mixed effects models adjusted for key demographic characteristics, health behaviors and body size. Epigenetic age metrics at the index visit were negatively correlated with the trajectory of muscle area over follow-up. Higher GrimAge at the index visit was associated with faster loss in muscle area and faster increase in fat areas (p < .05). Our analyses suggest that further exploration of DNAm with trajectories of muscle parameters may elucidate novel pathways of muscle dysfunction.

